# Quantum-aided secure deep neural network inference on real quantum computers

**DOI:** 10.1038/s41598-023-45791-z

**Published:** 2023-11-05

**Authors:** Hanqiao Yu, Xuebin Ren, Cong Zhao, Shusen Yang, Julie McCann

**Affiliations:** 1 National Engineering Laboratory for Big Data Analytics, Xi’an Jiaotong University, Xi’an, 710049 China; 2 Ministry of Education Key Laboatory for Intelligent Networks and Network Security, Xi’an Jiaotong University, Xi’an, 710049 China; 3 Department of Computing, Imperial College London, London, SW7 2AZ UK

**Keywords:** Quantum information, Computational science, Information technology

## Abstract

Deep neural networks (DNNs) are phenomenally successful machine learning methods broadly applied to many different disciplines. However, as complex two-party computations, DNN inference using classical cryptographic methods cannot achieve unconditional security, raising concern on security risks of DNNs’ application to sensitive data in many domains. We overcome such a weakness by introducing a quantum-aided security approach. We build a quantum scheme for unconditionally secure DNN inference based on quantum oblivious transfer with an untrusted third party. Leveraging DNN’s noise tolerance, our approach enables complex DNN inference on comparatively low-fidelity quantum systems with limited quantum capacity. We validated our method using various applications with a five-bit real quantum computer and a quantum simulator. Both theoretical analyses and experimental results demonstrate that our approach manages to operate on existing quantum computers and achieve unconditional security with a negligible accuracy loss. This may open up new possibilities of quantum security methods for deep learning.

## Introduction

Deep neural networks (DNNs) are machine learning models that have achieved impressive success across different domains such as science, medicine, humanities, and engineering, respectively^1–4^. Yet using a DNN model in the real world is often accompanied by security risks. The process of using a trained DNN model to make prediction is called DNN inference. Due to the complexity of computation, DNN inference services are predominantly deployed on the Cloud, bringing in the possibility of malicious attacks from the Cloud service provider and eavesdroppers. It is challenging to achieve information security for DNN inference considering its nature as a highly complex two-party computation process between the data holder (inference service consumer) and the model owner (Cloud service provider)^5–8^.

Methods based on classical cryptography like secure multi-party computing and homomorphic encryption have been introduced to secure DNN inference^5,8–12^. However, both in theory and practice, the existing methods are based on the restriction on the technology the attackers can use, as well as some unproved mathematical propositions^9,13^, and the advance of new algorithms and computation methods like quantum computing could potentially pose security vulnerability. The security weakness leads to the risk of data and DNN model leakage, and may bring concerns on applying DNNs in sensitive areas. Consequently, achieving unconditionally secure inference is desirable, but theoretically impossible using classical computation^14^.

Quantum cryptography and quantum computing technologies promote a whole new set of possibilities for unconditionally secure computation. Although it is demonstrated by the Mayers-Lo-Chau (MLC) no-go theorem^15,16^, that ideal one-sided two-party secure computation is impossible under both classical and quantum settings, several recent works have shown that similar effects can be achieved with quantum-based strategies by relaxing the restriction of ideal one-sided two-party secure computation, such as vector product^17^, delegated quantum computing^18–21^, and oblivious transfer^22–25^. Unlike the classical cryptographic methods, the security of such quantum methods are based on only fundamental physical laws rather than non-guaranteed algorithmic assumptions, therefore are unconditionally secure against all possible attacks^26^.

In this research, we bring the idea of quantum cryptography into DNN inference, and design a quantum-aided method for unconditionally secure DNN inference, overcoming the constraints of classical methods. Specifically, in our protocol, we guarantee that the following security requirements are satisfied without any assumptions on not only the classical but also the quantum computation ability of any possible attackers. First, the data from the data holder and the inference results are hidden from the model provider in the protocol. Second, information about the DNN model is hidden from the data holder except what can be logically inferred from the data and the inference results. Finally, no information from either party is leaked to the eavesdropper through the channels used in the protocol.

The basic idea is to first achieve a noisy version of secure quantum oblivious transfer (QOT), an universal primitive that can be used to compose arbitrary secure two-party computations^27^, with the help of an untrusted third party. Based on that, we can thereby compose unconditionally secure DNN inference. However, several challenges need to be resolved to make this practical. First, the oblivious transfer-based secure computing methods mostly rely on high-fidelity computing, that significantly hinders complex computations. In addition, a great number of oblivious transfer operations are required in general secure computations, but the quantum capacity of real quantum computers is seriously limited. In this Article, we design a coding and computing protocol for DNN inference that overcomes such limits in today’s quantum computers. Based on the intrinsic noise tolerance of DNNs^28,29^, a scheme is introduced into the DNN model training that enables DNN model to tolerate a high computation error rate during the inference. Thus, we relax the fidelity requirement of the QOT protocol, and consequently make our QOT protocol and secure DNN inference feasible on comparatively low-fidelity quantum computers with modest quantum capacity.

**Figure 1:**
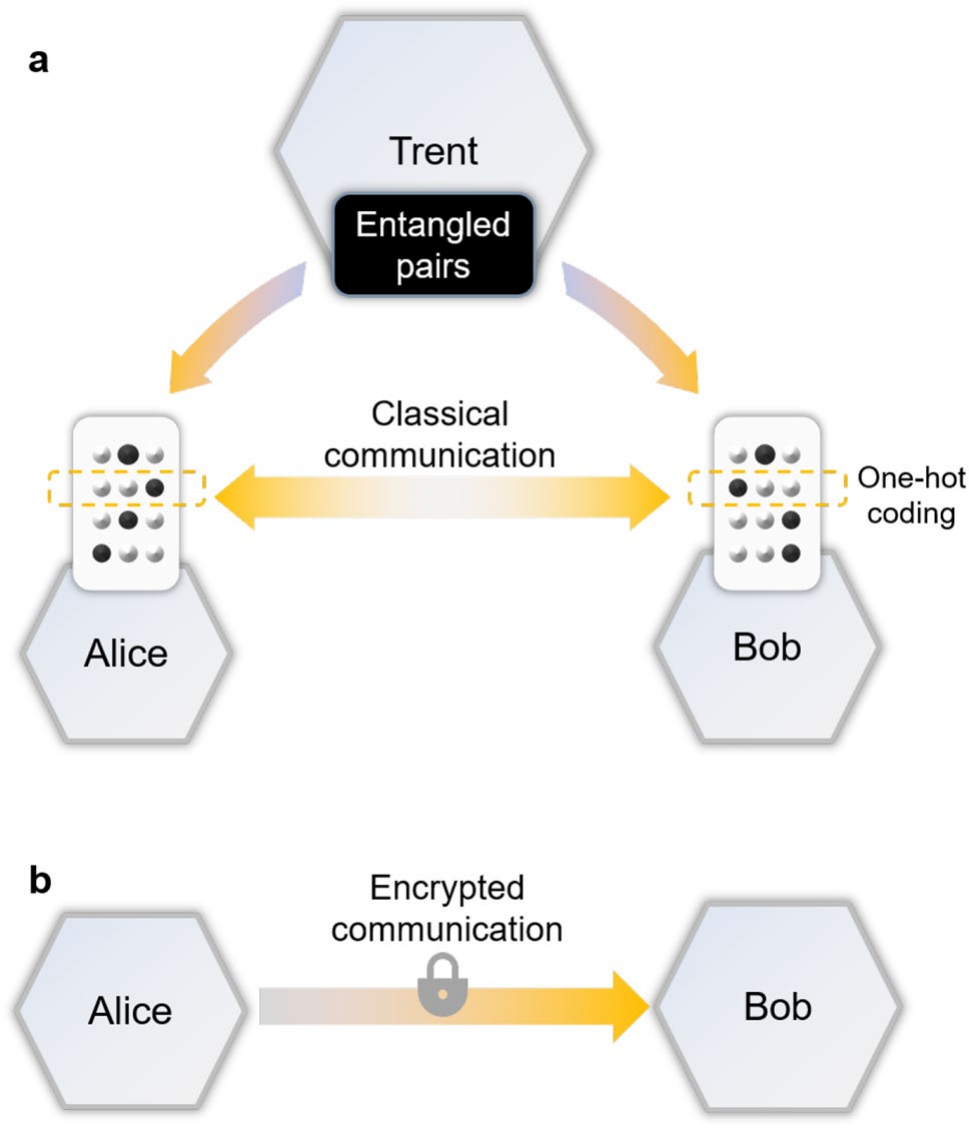
Securing DNN inference with (**a**) QOT and (**b**) classical two-party secure computing. In the secure inference with QOT, the data holder (Alice) and the model provider (Bob) collaborate by measuring the entangled pairs from the third party (Trent) and exchanging the index of some of their measurements, while in the classical case Alice sends encrypted data to Bob.

Here we introduce the system design for secure DNN inference based on QOT. The security of our method against classical and quantum adversaries is theoretically guaranteed as long as sufficient quantum capacity is available. The overall framework of the proposed design is shown in Fig. 1. A DNN, just like other artificial neural networks, consists of several layers of neurons, and the neurons are interconnected layer by layer^30,31^. This architecture can be modelled with a cascade of affine transformations followed by nonlinear activation functions. In our approach, the deep neural network is first split into several basic blocks such as vector addition and matrix multiplication. The blocks’ inputs and parameters are provided by the data holder and the model provider, respectively.

The blocks are evaluated with a stochastic protocol based on QOT to prevent unnecessary information revelation to either the data holder or the model provider. We design an algorithm and the corresponding coding to evaluate the basic blocks for DNN inference with an oblivious transfer primitive, and the quantum protocol to implement the oblivious transfer, which requires low quantum capacity and is suitable for fairly noisy quantum channels.

Finally, we demonstrate the effectiveness of the proposed approach for basic operators and DNN inference tasks through extensive experiments on the IBMQ quantum computer^32^. We also validate our method’s effectiveness on large DNNs using quantum simulators with several DNN models for different tasks, including general image and medical image classifications. We show that our approach enables secure inference for mainstream DNN models and common machine learning tasks.

## Results

### Quantum protocol for oblivious transfer

**Figure 2:**
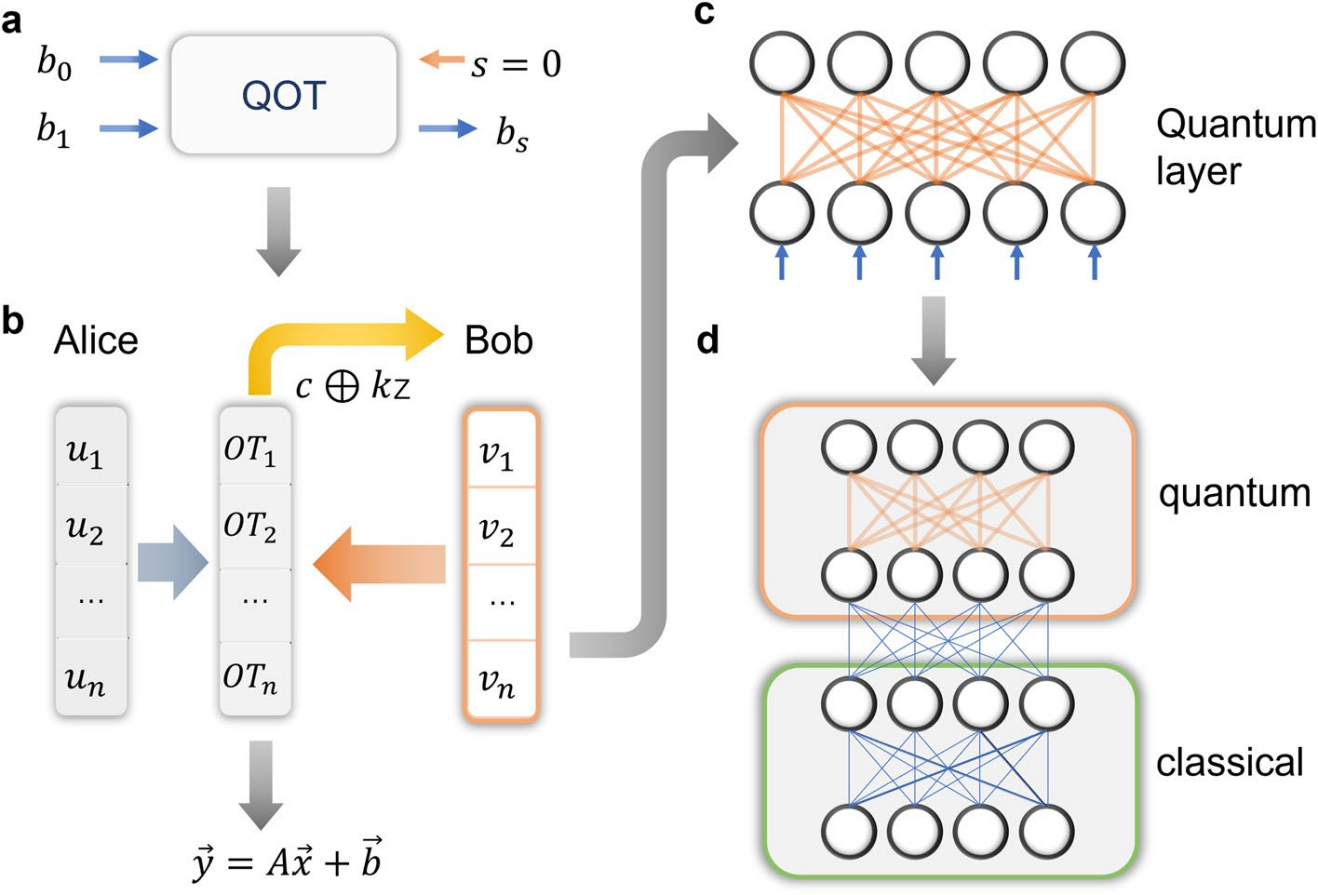
The architecture of quantum-aided secure DNN inference. (**a**) The basic component of quantum-aided secure DNN inference is QOT. (**b**) The basic operator is securely evaluated with QOT, and the affine transformation is composed with the basic operators. (**c**) The neural network is implemented with affine transformations as the basic blocks. (**d**) Complex DNNs are split into classical layers and quantum layers. Layers considered to be sensitive (e.g., the layer directly outputs the result) are implemented with QOT to avoid privacy leakage.

For DNN inference in an unconditionally secure manner in real world, a practical secure quantum cryptographic primitive has to be established first. Here we propose a quantum oblivious transfer (QOT) protocol that is applicable to commercially available quantum infrastructures with limited fidelity and quantum capacity, and provide a theoretical security guarantee. Figure 2a shows the schematic diagram of one-out-of-two oblivious transfer, a certain type of oblivious transfer, where a sender (say Alice) prepares and transfers two one-bit messages $b_0$ and $b_1$ to a receiver (say Bob). Bob can choose to learn either one of the two messages, $b_s, s \in \{0,1\}$, but learns nothing about the remaining one $b_{1-s}$. Obviously, Alice can also prepare two Bernoulli distributions $B_0$ and $B_1$, and send the samplings of these two distributions as messages.

The MLC no-go theorem implies that the ideal one-sided two-party oblivious transfer is impossible to be unconditionally secure, with either classical or quantum methods^14,16,33^. Hence, we adopt a three-party model where any party can be dishonest, but the third party cannot collude with the communicating parties. We will elucidate why this assumption does not violate the requirements of unconditional security. To achieve the concept of oblivious transfer, we refer to this third party as Trent.

In our method, Trent serves as a quantum state generator not directly involved in the computation. We first assume that Trent can operate the Hadamard gate $\mathtt H$, Toffoli gate $\texttt{CCNOT}$, and Pauli X gate $\mathtt X$, while Alice and Bob can measure the quantum state. During computing, we suppose that Trent can be fully dishonest but not collude with any of the other participants, which is a feasible setting, because the dishonesty of Trent’s can be detected by Alice and Bob with certain pre-agreed ways (see Supplementary Information S1). Trent only use public and unconditionally secure channels and therefore such checking will not affect the security. Note that all unidirectional communications of classical information are implemented in a strictly confidential manner that were strictly confirmed to be feasible with Quantum Key Distribution^26^.

**Figure 3:**
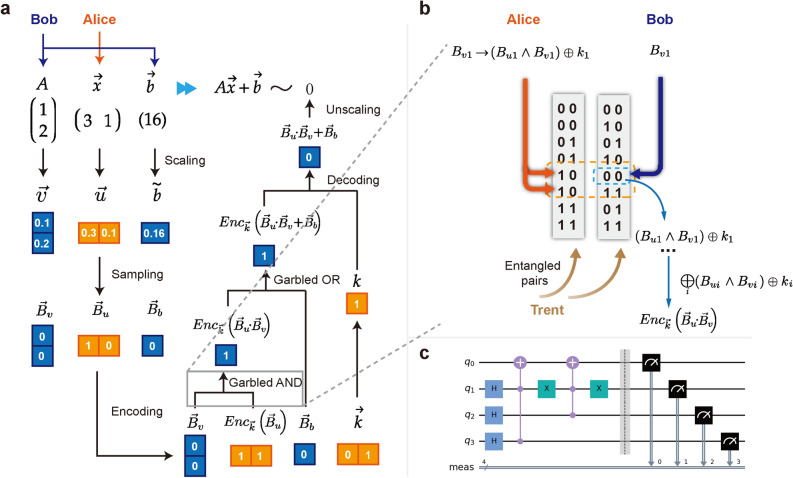
Overview of affine transformation based on QOT. (**a**) The flow chart of 2-dimensional affine transformation. First, Alice prepares input $\vec {x}$ and Bob prepares *A* and $\vec {b}$. Then the matrices are scaled so that the norms of matrices are lower than 1, and are sampled as input binary matrices. The secure affine transformation is conducted through encoding and encoded operations based on QOT. (**b**) The diagram of encoded AND gate implementation based on QOT. Trent sends entangled pairs. Alice and Bob perform the encoded AND by transmitting the indices. (**c**) The quantum circuit for Trent to prepare entangled pairs.

The entire process can be divided into three stages: state preparation, validation and transfer. For the state preparation stage, Trent prepares a sequence of entangled quantum states and sends the entangled pairs to Alice and Bob. First, Trent generates a sequence of identical states $\left\{ \mathinner {|{\psi _{ab}}\rangle }\right\}$ in the state space for four qubits ${\mathscr {H}}$, such that each state satisfies

1 $$\begin{aligned} \mathinner {|{\psi _{ab}}\rangle } = \sum _{b_0=0}^1{\sum _{b_1=0}^1{\sum _{s=0}^1{\mathinner {|{2 b_1 + b_0}\rangle }\otimes \mathinner {|{s, b_s}\rangle }}}},  \end{aligned}$$

where a 4-qubit quantum state is written as summation of tensor products of pairs of two-qubit quantum states. The first part is represented using the corresponding binary values (e.g., $\mathinner {|{1}\rangle }\otimes \mathinner {|{1}\rangle }$ is written as $\mathinner {|{3}\rangle }$), while the second part is depicted using conventional notation (e.g., $\mathinner {|{1}\rangle }\otimes \mathinner {|{1}\rangle }$ is written as $\mathinner {|{1,1}\rangle }$). The quantum circuit to generate such a state is shown in Fig. 3b. Each state is split into two sub-states

2 $$\begin{aligned} \mathinner {|{\psi _{ab}}\rangle } = \mathinner {|{\psi _{a}}\rangle }\otimes \mathinner {|{\psi _{b}}\rangle }, \end{aligned}$$

where $\mathinner {|{\psi _{a}}\rangle } \in \mathscr {H}_a$, $\mathinner {|{\psi _{b}}\rangle } \in \mathscr {H}_b$, and $\mathscr {H}_a$, $\mathscr {H}_b$ are two-dimensional subspaces of $\mathscr {H} = \mathscr {H}_a\otimes \mathscr {H}_b$. Then, Trent sends the entangled states $\mathinner {|{\psi _{a}}\rangle }$, $\mathinner {|{\psi _{b}}\rangle }$ to Alice and Bob, respectively, and repeats this process for *n* times until both Alice and Bob separately get a sequence of quantum states. In this stage, the decoy bits technique^34^ is applied to prevent eavesdropping by outside attackers, which is achieved by inserting decoy particles randomly selected in $\mathinner {|{0}\rangle }$, $\mathinner {|{1}\rangle }$, $\mathinner {|{+}\rangle }$, and $\mathinner {|{-}\rangle }$ into the particles prepared for sending to Alice and Bob. Trent will then publish the insertion location and the measurement bases of the decoy particles. If an eavesdropper try to measure the states sent by Trent, some of the decoy particles will not be at the eigenstates of the measurement bases, and this will change the states of the decoy particles. Then Alice and Bob will find that the states of the decoy particles do not match the expected results, and the eavesdropper will be detected. After that, the decoy particles are discarded for the next stage.

For the validation stage, Alice and Bob receive the corresponding states and randomly choose some of the states for validation. Alice and Bob measure the bits of $\mathinner {|{\psi _{ab}}\rangle }$ chosen for validation with thewith the Pauli $\mathtt Z$ matrix ($\mathinner {|{1}\rangle }\mathinner {\langle {1}|} - \mathinner {|{0}\rangle }\mathinner {\langle {0}|}$). Note that the four bits should follow the one-out-of-two oblivious transfer relationship among $b_0$, $b_1$, *s*, and $b_s$. Alice and Bob share the indices of states for validation, and exchange the measurement results of the states they both selected for validation. If the portion of results following the one-out-of-two oblivious transfer relationship is less than a pre-agreed threshold based on the channel noise, Alice and Bob would find the protocol to be unreliable and abort the protocol. Otherwise, Alice and Bob will preserve a sub-sequence of the quantum states that none of them selected for validation, for the next stage. We denote such sub-sequences Alice and Bob kept as $S_a = \left\{ \mathinner {|{\psi _{a}}\rangle }^i\right\} _{i=0}^n$ and $S_b = \left\{ \mathinner {|{\psi _{b}}\rangle }^i\right\} _{i=0}^n$, respectively.

The final stage is transfer, where Bob measures the quantum states in $S_b$ and saves the indices of states whose first bit is $s, \,s\in \{0,1\}$. The index set of states chosen by Bob is denoted as $I_b$. If $I_b$ is empty, Bob claims the process has failed and all parties start over from the state preparation stage. Otherwise, Bob sends $I_b$ to Alice. Alice measures the states in $S_a$ at the indices in $I_b$, and stores the indices where the measurement result is equal to $2 b_1 + b_0$ as $I_a$. Finally, Alice randomly chooses an index $i_a$ in $I_a$ and sends it to Bob. The second bit of Bob’s measurement at position $i_a$ is the output of the QOT process. The overall process is depicted in Fig. 2b and introduced in more detail in the Supplementary Information S1.

The output bit is the QOT output for the following reason. Denoting the measurements of $i_a$-th sub-states as $M^{i_a}_{a1}$, $M^{i_a}_{a2}$, $M^{i_a}_{b1}$, and $M^{i_a}_{b2}$, respectively, we have $M^{i_a}_{b1} = s$ and $2 M^{i_a}_{a1}+M^{i_a}_{a2}=2 b_1 + b_0$. According to Eq. (1), we have $M^{i_a}_{b2} = b_s$.

We claim that QOT is unconditionally secure, because neither Alice nor Bob can interfere with Trent’s state generations, making attacks from Alice or Bob impossible. Meanwhile, as the measurement results of Bob’s or Alice’s alone contain no secret information, Trent gets no information by attacking Alice or Bob. The formal security proof is demonstrated in Methods.

Note that, since neither our protocol itself nor its security proof depends on the low error rate assumption, QOT can tolerate high error rates (noise levels) in quantum computing and quantum communication. Particularly, our protocol passes the error in quantum computing and quantum channels to the next step for DNNs to deal with. Therefore, the overall noise tolerance level only depends on the noise tolerance of DNNs, which can be set by manually introducing noises during DNN training. This is explicitly discussed in Methods.

### Implementing deep neural networks with quantum-aided blocks

To compose a DNN model with the QOT primitive, the basic blocks of DNN models have to be implemented first. As shown in Fig. 2b, the basic DNN blocks are affine transformations naturally composed of vector inner product and vector addition

3 $$\begin{aligned} A\vec {x} + \vec {b} = \left( \vec {a}_1\cdot \vec {x}, \vec {a}_2\cdot \vec {x}, \ldots , \vec {a}_m\cdot \vec {x}\right) + \vec {b} \end{aligned}$$

where $A = \left( \vec {a}_1^T, \vec {a}_2^T, \ldots , \vec {a}_m^T\right)$. Theoretically, a QOT protocol enables us to conduct certain kinds of secure two-party computation with a noisy channel. Here we show how to implement secure vector inner product with our QOT protocol, and the implementation of secure vector addition is shown in Methods. The process contains encoding, secure computation, and decoding. All unidirectional communications are assumed to be strictly confidentially.

Say Alice holds a vector $\vec {u} = \left( u_1, u_2, \ldots , u_m\right)$ such that $u_i >0,\, \sum {u_i}<1,\, i=1,2,\ldots ,m$, and Bob holds $\vec {v} = \left( v_1, v_2, \ldots , v_m\right)$ such that $v_i > 0,\,\sum {v_i}=\gamma < 1$. Each of the vectors corresponds to a categorical distribution of a one-hot binary vector. For example, we have an *m*-dimensional binary vector $\vec {B}_u = \left( 0, 0, \ldots , 1, \ldots , 0\right)$, $P\left( B_{uk}=0\right) = u_k, k=1,2,\ldots ,m$. Similarly we have $\vec {B}_v = \left( 0, 0, \ldots , 1, \ldots , 0\right)$, $P\left( B_{vk}=0\right) = v_k, k=1,2,\ldots ,m$ and $P\left( \vec {B}_v=\vec {0}\right) = 1-\gamma$.

Treating the computation of $\vec {u}\cdot \vec {v}$ as an example, for encoding, Alice first samples a binary vector $\vec {b}_a$ according to $\vec {u}$ and so does Bob. Alice encodes the binary vector with a random binary one-time pad $\vec {k}$,

4 $$\begin{aligned} Enc_{\vec {k}}\left( \vec {b}_u\right) = \left( B_{u1}\oplus k_1, B_{u2}\oplus k_2, \ldots , B_{um}\oplus k_m\right) . \end{aligned}$$

Then Alice prepares a sequence of encoded $\texttt{AND}$ gates with QOT $\left( OT_1, OT_2, \ldots \right)$ to compare Alice’s encoded binary vector with Bob’s, where $OT_n$ represents a oblivious transfer operation that returns the corresponding value. Each encoded $\texttt{AND}$ takes encoded bits as input and outputs the encoded results, which follows

5 $$\begin{aligned} OT_i\left( 0\right) = 0\oplus k_i, \; OT_i\left( 1\right) = B_{ui}\oplus k_i. \end{aligned}$$

For secure computation, Bob evaluates the sequence of the oblivious transfer gates with his own binary vector $\vec {b}_b$ and gets

6 $$\begin{aligned} \begin{aligned} \vec {c}&= \left( OT_1\left( B_{v1}\right) , OT_2\left( B_{v2}\right) , \ldots , OT_m\left( B_{vm}\right) \right) \\&= \left( \left( B_{u1}\wedge B_{v1}\right) \oplus k_1, \left( B_{u2}\wedge B_{v2}\right) \oplus k_2, \ldots , \left( B_{um}\wedge B_{vm}\right) \oplus k_m\right) . \end{aligned} \end{aligned}$$

Bob’s output is obtained with an exclusive-or computation $\bigoplus$ (exclusive or) on $\vec {c}$. Similarly, Alice computes the decoding key with $\bigoplus$ on $\vec {k}$. Bob’s and Alice’s results are respectively given as

7 $$\begin{aligned} c = \bigoplus _{i=1}^m {c_i}, \; k = \bigoplus _{i=1}^m {k_i}. \end{aligned}$$

The final output of secure computation is given by

8 $$\begin{aligned} \begin{aligned} k\oplus c&= k\oplus \bigoplus _{i=1}^m {\left( B_{ui}\wedge B_{vi}\right) \oplus k_i}\\&= k\oplus \bigoplus _{i=1}^m {B_{ui}\wedge B_{vi}} \oplus \bigoplus _{i=1}^n {k_i} = \bigoplus _{i=1}^m {B_{ui}\wedge B_{vi}}. \end{aligned} \end{aligned}$$

For decoding, the final multiplication result is obtained either by Alice sending *k* to Bob or by Bob sending *c* to Alice. The inner product, as the final computation object, $\vec {u}\cdot \vec {v}$ is given by the probability below.

9 $$\begin{aligned} \begin{aligned} P\left( k\oplus c = 1\right)&= P\left( \bigoplus _{i=1}^m {B_{ui}\wedge B_{vi}} = 1\right) = \vec {u}\cdot \vec {v}, \end{aligned} \end{aligned}$$

implying that we can sample the binary value of the vector inner product with this process. A single binary evaluation is called a shot, and a more accurate result can be obtained by repeating the process above for more shots. More details about the implementation of other operators for DNNs are demonstrated in Methods. An outline of a two-dimensional affine transformation based on QOT is shown in Fig. 3.

According to the discussion above, we have the basic building blocks for secure deep learning via QOT. These provide us with an operator set for DNNs, and the next step is to set up a neural network with such operators. The general architecture of quantum-aided DNNs is shown in Fig. 2, which is divided into the operator and network layers. Figure 2a,b show the operator layer of quantum-aided DNNs. First, QOT and quantum secure communication make up the basic operator set, including secure inner product and secure addition. By composing the basic operators, we have an operator set consisting of affine transformations. Figure 2c,d show the network layer of quantum-aided DNNs. A DNN can be comprised of affine transformation blocks. Some layers can remain to be evaluated with classical computing for speeding up, and the rest are evaluated with the quantum protocol to ensure security.

### Simulation and experiment results

We implemented the QOT gate on both real quantum computers on the Cloud and noisy classical quantum simulators. In this Article we used a quantum computer from the IBM Quantum Experience Program^35^ to validate QOT’s core characteristics, including the introduced noise. The error rate for a single QOT operation is 0.179, and more details are given in Methods. Additionally, the error rate can be further reduced with an application-specific quantum computer like a photon computer.

**Figure 4:**
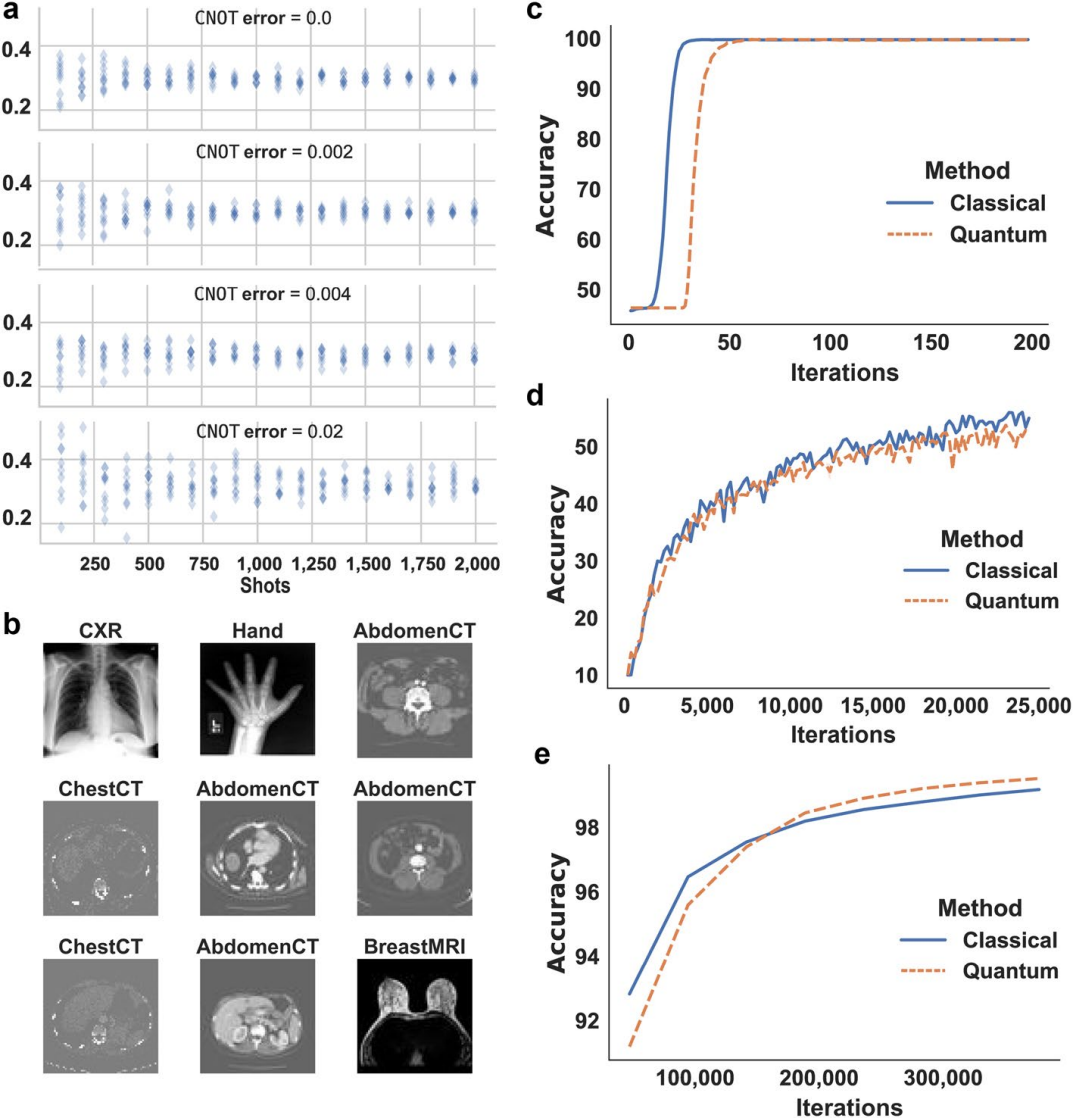
The experiment results on real quantum computer and simulator. (**a**) The computation results for QOT-based vector product. (**b**) Classification results of medical images in MedNIST dataset. (**c**–**e**) The classification accuracy curves on MNIST, CIFAR-10 and MedNIST, respectively.

Firstly the computation error of basic blocks was evaluated. Figure 4a illustrates the products of three-dimensional vector multiplication using simulated quantum systems with different $\texttt{CNOT}$ error levels, where the ideal product is 0.3. It is obvious that the products disperse as the $\texttt{CNOT}$ error increases, and converge to the ideal product when the shot number increases. To achieve an acceptable product error, we take 2000 shots of evaluations for each DNN inference to balance the resource usage and accuracy. The error rate also limits the scale of affine transformations. Although the product error can be corrected by adjusting parameter $\lambda$ (see Methods), a higher error rate does introduce higher noise to the result. Specifically, we applied an affine transformation with five-dimensional inputs as the basic secure operator in the experiment.

For real quantum computer validations, we used a fully connected neural network for the binary MNIST classification task^36^, where the model was trained on 12,000 handwritten 0 and 1 digits and validated on 2000 digits. Our model comprised an input layer with 784 ($28\times 28$) neurons, three hidden layers with 512, 128 and five neurons respectively, and a $5\times 2$ fully connected output layer. The output layer was implemented with the quantum protocol. The model was trained with classical backpropagation and tested (inference) with quantum-aided evaluation. According to Fig. 4c, the classical-quantum hybrid model identifies the digits without a noticeable classification accuracy loss.

We also conducted extensive simulations with the Qiskit linear-algebra-based simulator to demonstrate our protocol’s applicability to larger DNNs with special-purpose quantum infrastructures. The noise was imported so that the final oblivious transfer error rate was $5\times 10^{-3}$, under which the input width of the neural network can be up to 100. We used a modified AlexNet^37^ to classify the CIFAR-10 dataset^38^ in simulations, which is a common image classification benchmarking setting. The modified AlexNet consists of five convolutional layers and three fully connected layers with widths 100, 84, and 10, respectively. The last two layers were implemented with the quantum protocol. The classical-quantum hybrid model was trained for 10 epochs when the accuracy converged. The accuracy of the quantum and classical models is compared in Fig. 4d and the result is also summarized in Table 1, implying that the quantum-aided model brings little accuracy loss (less than 2%).

**Table 1:** The accuracy of quantum-aided DNNs compared with classical DNNs.

Classification datasets	Classical DNN (%)	Quantum-aided DNN (%)
Binary MNIST	99.85	99.68
CIFAR-10	54.20	52.62
MedNIST	99.17	99.51

To further validate our approach on real-world sensitive data, we conducted simulations on the common dataset for medical image classification, MedNIST^2^. The noise was imported so that the $\texttt{CNOT}$ error rate was $5\times 10^{-2}$, comparable to that of the available quantum infrastructure^39^. A modified AlexNet with two convolutional layers and four fully connected layers with widths 120, 84, 12 and 6, respectively was adopted as the classifier. The last two layers were implemented with the quantum-aided protocol. The model was trained for 10 epochs. The classification results and accuracy curve are illustrated in Fig. 4b,e, demonstrating that our method has comparable performance with the classical DNN model on real medical images. Experimental results above are summarized in Table 1, showing that the loss brought by our quantum protocol is insignificant ($\le 1.58\%$) in these tasks.

**Figure 5:**
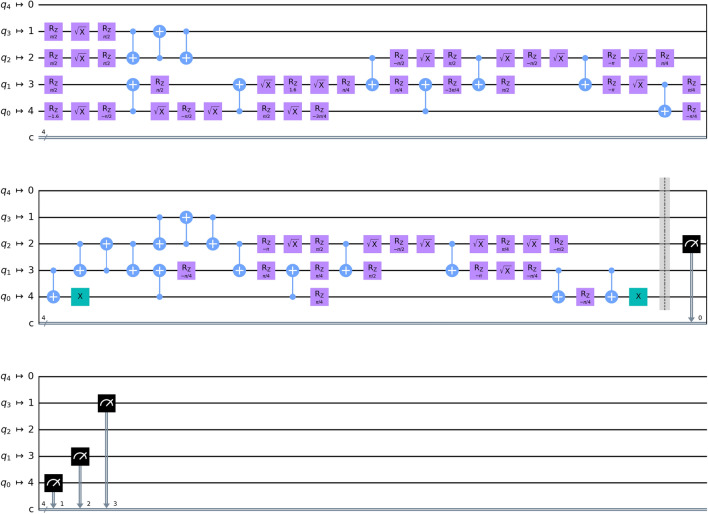
The implementation of the quantum oblivious transfer circuit.

## Discussion

In summary, we propose a methodology for secure DNN inference augmented by quantum technology, utilizing commercially available quantum computing infrastructures. Our approach introduces a classical-quantum hybrid architecture to implement DNNs while ensuring secure inference. Notably, we present a Quantum Oblivious Transfer (QOT) protocol that has been proven to be unconditionally secure, forming the basis for a fundamental set of operators supporting secure DNN inference.

In principle, our work demonstrates the advantages of quantum information technologies in achieving unconditional security for DNN inference, primarily by involving an untrusted third party. However, it’s important to note that the fidelity of quantum computing and quantum channels can impact the efficiency of our method. This challenge may be particularly pertinent when applying our approach to large DNN models using commercially available quantum infrastructures^31^.

Future research and development efforts will enable the extension of our methodology to handle larger and more complex DNNs with additional layers and diverse operators. Ultimately, our work represents an exciting initial step towards achieving unconditionally secure deep learning, offering promising prospects for the intersection of quantum technology and machine learning security.

## Methods

### Implementation of quantum oblivious transfer

We implemented our quantum circuit with the Qiskit framework and the ibmq_santiago cloud quantum computer provided by IBM Quantum Experience. The ibmq_santiago has five qubits, which is sufficient for our protocol which requires four qubits, and the average error rate of $\texttt{CNOT}$ gate is $6.746\times 10^{-3}$. The Toffoli gate used in the quantum circuit was implemented with the single-bit quantum gates and the $\texttt{CNOT}$ gates. The decomposition of the proposed QOT circuit is shown in Fig. 5. For both real quantum computer and quantum simulator, the quantum circuit was built and executed with the Qiskit Python quantum programming framework^40^.

### Security of quantum oblivious transfer

In this part, we demonstrate that the proposed Quantum Oblivious Transfer (QOT) protocol remains secure against any malicious adversaries, as long as Trent does not engage in collusion with any party. We begin by assuming the confidentiality of quantum channels among all parties, employing established techniques such as decoying and privacy amplification for securing quantum channel establishment^34^. These security measures can be implemented through the use of quantum decoy particles or via quantum teleportation across a confidential classical channel^34,41^. Notably, we emphasize that classical communication between Alice and Bob is kept confidential. As a result, any physical channel connecting arbitrary parties does not lead to information leakage, rendering the need for further discussion on external attacks unnecessary.

Moreover, as eavesdropping cannot yield any more valuable information, collusion between participants and an eavesdropper is tantamount to an attack by a single participant.

Regarding attacks from participants, we first consider Trent’s attempt to pilfer information. Trent may act dishonestly and deviate from the protocol by preparing states entangled with Trent’s personal state $\mathinner {|{f\left( b_1,b_0, s\right) }\rangle }$:

10 $$\begin{aligned} \sum _{b_0=0}^1{\sum _{b_1=0}^1{\sum _{s=0}^1{\mathinner {|{b_1,b_0, s, b_s}\rangle }\otimes \mathinner {|{f\left( b_1,b_0, s\right) }\rangle }}}} . \end{aligned}$$

In this case, Trent might gain access to Alice’s or Bob’s measurement results. However, Trent can only ascertain the exact values of $b_0$, $b_1$, or *s* if and only if Trent possesses knowledge of indices $I_a$ ($I_b$). Nevertheless, the transmission of states in the QOT protocol is presumed to occur via strictly confidential channels, such as a one-time pad with quantum key distribution. Consequently, Trent can glean no information about Alice’s or Bob’s private data, but only a sequence of random bits resulting from Trent’s entanglement attack.

Next, we consider the possibility of an attack from either Alice or Bob attempting to intercept quantum communication between the other party and Trent. However, this scenario is equivalent to an external attack, which has already been demonstrated to be infeasible above. The sole information available is derived from $I_b$ or $i_a$. Importantly, for $c \in \left\{ 0,1\right\}$, the conditional probabilities $P\left( s=c|I_b\right)$ and $P\left( b_{s-1}=c|I_b,i_a\right)$ consistently hold at $\frac{1}{2}$, ensuring that no unnecessary information leaks from $I_b$ or $i_a$.

### Secure evaluation for basic blocks of DNNs

The secure evaluation of matrix multiplication is introduced in Results. Here we introduce the secure evaluation for vector addition.

The process is similar to secure vector multiplication. Suppose that Alice holds an *m*-dimensional vector $\vec {u}$ such that $u_i > 0,\sum {u_i}=\gamma < 1$, and similarly Bob holds $\vec {v}$ with the same property. In the encoding stage, Alice samples a binary vector $\vec {b}_a$ according to $\vec {u}$ and so does Bob in the same way of secure vector multiplication. Bob’s vector is encoded with a binary one-time pad $\vec {k_b}$ that is only known to Bob,

11 $$\begin{aligned} Enc_{\vec {k_b}}\left( \vec {B}_b\right) = \left( B_{b1}\oplus k_{b1}, B_{b2}\oplus k_{b2}, \ldots , B_{bm}\oplus k_{bm}\right) . \end{aligned}$$

Alice also holds a secret one-time pad $\vec {k_a}$, then prepares a sequence of encoded $\texttt{OR}$ gates to simulate the addition with QOT $\left( OT_1, OT_2, \ldots , OT_m\right)$. Each encoded $\texttt{OR}$ gate follows

12 $$\begin{aligned} \begin{aligned} OT_i\left( 0\right)&= \left( \left( 0\oplus k_{bi}\right) \vee {\tilde{B}}_{ai}\right) \oplus k_{ai},\\ OT_i\left( 1\right)&= \left( \left( 1\oplus k_{bi}\right) \vee {\tilde{B}}_{ai}\right) \oplus k_{ai}. \end{aligned} \end{aligned}$$

In the secure computing stage, Bob evaluates the sequence of the oblivious transfer gates with his own binary vector and gets

13 $$\begin{aligned} \begin{aligned} \vec {c}&= \left( OT_1\left( B_{b1}\right) , OT_2\left( B_{b2}\right) , \ldots , OT_m\left( B_{bm}\right) \right) \\&= \left( \left( B_{a1}\vee B_{b1}\right) \oplus k_{a1}, \left( B_{a2}\vee B_{b2}\right) \oplus k_{a2}, \ldots \left( B_{am}\vee B_{bm}\right) \oplus k_{am}\right) . \end{aligned} \end{aligned}$$

In the final decoding stage, Alice could either send the key $\vec {k_a}$ to Bob to reveal the computation result, or keep the output encoded as the input for the next secure operator.

As mentioned above, in both addition and multiplication setting, the vectors are required to be non-negative, and the L1-norm of the vector should not exceed $\gamma$, which is not mathematically complete for building a general neural network. However, a common neural network can be built with limited operators without losing accuracy using weight clamping and scaling^42^, which is applied in this Article.

### The impact of QOT noise for inference

Here the impact of noise in QOT for inference is analyzed. As long as the oblivious transfer error rate $\epsilon$ satisfies $2m\epsilon \ll 1$, the final approximate result follows (see Supplementary Information S1)

14 $$\begin{aligned} P\left( k\oplus c = 1\right) = \left( 1-\lambda \right) \vec {u}\cdot \vec {v} + \lambda \left( 1-\vec {u}\cdot \vec {v}\right) , \end{aligned}$$

where $\lambda$ is the computation error rate, which follows

15 $$\begin{aligned} \lambda = \frac{1}{2} - \frac{1}{2}\left( 1-2\epsilon \right) ^m. \end{aligned}$$

Thus, the corrected final result follows

16 $$\begin{aligned} \vec {u}\cdot \vec {v} = \frac{P\left( k\oplus c = 1\right) - \lambda }{1-2\lambda }. \end{aligned}$$

Usually, such a probabilistic approximation can bring substantial noise to computation. However, due to the noise tolerance of DNNs, the computation errors incur little to no degradation of accuracy as long as the noise caused by computation errors is below a threshold. Assuming that the maximum noise tolerance of the DNN layer after the quantum-aided block is given in the form of max variance $\sigma _{max}$ of the noise, the upper bound of the computation error rate is (see Supplementary Information S1)

17 $$\begin{aligned} \lambda < \frac{1}{2} - \frac{1}{4\sqrt{n}\sigma _{max}}. \end{aligned}$$

During the training of the DNN model, first, a noise tolerance requirement is estimated according to Eq. (17). Then the corresponding Gaussian noise is added to the quantum-aided blocks of the DNN model to enhance the noise tolerance of DNNs^43^. The trained DNN model is tolerant to noise lower than the additional noise, which guarantees that the DNN model can handle the noise brought by quantum errors with a lower level in terms of standard deviation of noise.

### Supplementary Information

Supplementary Information.

## Data Availability

All data used in this work are publicly available through online sources as follows: the MNIST dataset^36^ (https://www.kaggle.com/datasets/hojjatk/mnist-dataset), the CIFAR-10 dataset^38^ (https://www.cs.toronto.edu/~kriz/cifar.html) and the MedNIST dataset^2^ (https://github.com/apolanco3225/Medical-MNIST-Classification).
