# Author Correction: Quantum-aided secure deep neural network inference on real quantum computers

**DOI:** 10.1038/s41598-024-55484-w

**Published:** 2024-03-11

**Authors:** Hanqiao Yu, Xuebin Ren, Cong Zhao, Shusen Yang, Julie McCann

**Affiliations:** 1 National Engineering Laboratory for Big Data Analytics, Xi’an Jiaotong University, Xi’an, 710049 China; 2 Ministry of Education Key Laboatory for Intelligent Networks and Network Security, Xi’an Jiaotong University, Xi’an, 710049 China; 3 Department of Computing, Imperial College London, London, SW7 2AZ UK

Correction to: *Scientific Reports* 10.1038/s41598-023-45791-z, published online 05 November 2023

The original version of this Article contained errors in the Acknowledgements section.

 “We thank M. Kim, T. Haug, A. Smith and C. Ling for the feedback on this work. We acknowledge grants as follows: this work was supported in part by the National Key Research and Development Program of China under Grant 2020YFA0713900; the National Natural Science Foundation of China under Grants 61772410, 61802298, 62172329, U1811461, U21A6005, 11690011, and the China Postdoctoral Science Foundation under Grants 2020T130513, 2019M663726.”

now reads:

“We thank M. Kim, T. Haug, A. Smith, and C. Ling for the feedback on this work. We acknowledge grants as follows: this work was supported in part by the National Key Research and Development Program of China under Grants 2021YFB2401300, 2022YFA1004100, and 2020YFA0713900; in part by the National Natural Science Foundation of China under Grants 61772410, 61802298, 62172329, U1811461, U21A6005, and 11690011.”

The original Article has been corrected.

