# Supplementary material for: Quantum-aided secure deep neural network inference on real quantum computers
Source: Sci Rep. 2023 Nov 5;13:19130. doi: 10.1038/s41598-023-45791-z (PMC10625985; doi:10.1038/s41598-023-45791-z)
Supplement: Supplementary file 1 — Supplementary Information. [file 41598_2023_45791_MOESM1_ESM.pdf]

Supplementary Information for: Quantum-aided Secure Deep Neural Network Inference on Real Quantum Computers

Hanqiao Yu[1,+], Xuebin Ren[1,+,*], Cong Zhao[1,+], Shusen Yang[1,2,*], and Julie McCann[3,*]

[1]National Engineering Laboratory for Big Data Analytics, Xi'an Jiaotong University, 710049 Xi'an, China
[2]Ministry of Education Key Laboatory for Intelligent Networks and Network Security, Xi'an Jiaotong University, 710049 Xi'an, China
[3]Department of Computing, Imperial College London, SW7 2AZ London, U.K.
[*]xuebinren@mail.xjtu.edu.cn, shusenyang@mail.xjtu.edu.cn, j.mccann@imperial.ac.uk
[+]these authors contributed equally to this work

March 28, 2023

1 Preliminaries on Quantum Oblivious Transfer Protocol

The precise concept and definition of quantum oblivious transfer problem are given here, along with all necessary notations, definitions and analyses for reading the following contents.

Definition 1 (Quantum oblivious transfer)**.** *Alice holds two one-bit messages* b_0 *and* b_1 *and Bob holds a selection bit* $s \in \{0, 1\}$*. At the end of the protocol, Bob learns either one of the two messages* b_s *while knowing nothing about* b_{s-1}*, and Alice knows nothing about* s*.*

Definition 2 (Transversal operations)**.** *The following gates are used in our protocol:*

1. *Pauli-X gate:* $\mathtt{X} = |1\rangle\langle 0| + |0\rangle\langle 1|$,
2. *Pauli-Z gate:* $\mathtt{Z} = |1\rangle\langle 1| + |0\rangle\langle 0|$,

3. *Hadamard gate:* $\mathtt{H} = |+\rangle\langle 0| + |-\rangle\langle 1|$,

4. *Controlled not gate:* $\mathtt{CNOT} = |a\rangle|b\rangle \mapsto |a\rangle|a \oplus b\rangle$,

5. *Toffoli:* $\mathtt{CCNOT} = |a\rangle|b\rangle|c\rangle \mapsto |a\rangle|b\rangle|ab \oplus c\rangle$,

where $|+\rangle \equiv (|1\rangle + |0\rangle)/\sqrt{2}$ *and* $|-\rangle \equiv (|1\rangle - |0\rangle)/\sqrt{2}$ *are the eigenstates of particles spinning along the x-direction.*

2 Quantum Oblivious Transfer Scheme

For clarity, we assume Alice, Bob, and Trent are the participants. Alice has two one-bit messages b_0 and b_1, and Bob has a selection bit $s \in \{0,1\}$. Bob wants to know the result b_s with the help of the third party Trent, who might be malicious but does not collude with the other participants during the protocol. At the end of the process, Bob learns nothing except for the result b_s, and Alice learns nothing.

The steps of Quantum Oblivious Transfer (QOT) are listed below.

Input:	Alice holds two one-bit messages b_0 and b_1 and Bob holds a selection bit $s \in \{0,1\}$.
Output:	Bob $\leftarrow b_s$ and Alice and Trent get nothing.
Step 1:	Alice and Bob establish a confidential channel with the quantum key distribution.
Step 2:	Trent prepares n entangled four-bit systems given as follow,

$$|\psi_{ab}\rangle = \sum_{b_0=0}^{1}\sum_{b_1=0}^{1}\sum_{s=0}^{1} |2b_1 + b_0\rangle \otimes |s, b_s\rangle. \tag{1}$$

where n is a positive integer depending on the success rate of a single transmission. After that, Trent splits each state to two substates:

$$|\psi_{ab}\rangle = |\psi_a\rangle \otimes |\psi_b\rangle, \tag{2}$$

where $|\psi_a\rangle \in \mathcal{H}_a$, $|\psi_b\rangle \in \mathcal{H}_b$, and $\mathcal{H}_a$, $\mathcal{H}_b$ are two-dimensional subspaces of $\mathcal{H} = \mathcal{H}_a \otimes \mathcal{H}_b$. Then Trent arranges the states into two sequences of substates:

$$S_a = \left\{|\psi_a\rangle^i\right\}_{i=0}^{n}, \tag{3}$$

$$S_b = \left\{|\psi_b\rangle^i\right\}_{i=0}^{n}. \tag{4}$$

Trent also prepares several pairs of decoy particles randomly selected in $|0\rangle$, $|1\rangle$, $|+\rangle$, and $|-\rangle$ and randomly inserts them into S_a and S_b. Finally, Trent sends the mixed sequences S'_a and S'_b to Alice and Bob, respectively.

Step 3: Alice and Bob receive S'_a and S'_b, respectively. In the case that Alice and Bob have quantum storage ability, the sequences will be stored until Trent declares the positions of the decoy particles insertions, as well as the corresponding measurement operators of the particles (*e.g.*, $\mathtt{Z}$ for $|0\rangle$, $|1\rangle$ and $\mathtt{X}$ for $|+\rangle$, $|-\rangle$). Alice and Bob then extract the decoy particles in sequences S'_a and S'_b and measure them with the corresponding measurement operators. Thereafter Trent publishes the initial states of the decoy particles for Alice and Bob to compare their measurement results with the initial states and evaluate the error rate.

In the case that Alice and Bob have no quantum storage ability, Alice and Bob will respectively measure a certain percentage of particles with random bases. When Trent publishes the positions of the decoy particles, Alice and Bob will check the results that are included in the decoy particles and measured with the correct bases to evaluate the error rate. For conciseness, in the following discussion, we assume that Alice and Bob have quantum storage ability, since the quantum storage ability does not affect the security of the protocol.

If the error rate exceeds a certain threshold set in advance, they will take the protocol as insecure and abort the protocol. Otherwise, they will continue the protocol.

Step 4: Alice and Bob discard the decoy particles and recover the original sequences S_a and S_b, after which Alice measures each two-bit substate Alice received with $\mathtt{Z} \otimes \mathtt{Z}$. The sequence of measurement results is denoted as $M_a = \{(M_{a1}^1, M_{a2}^1), (M_{a1}^2, M_{a2}^2), \cdots (M_{a1}^n, M_{a2}^n)\}$. In the same way, Bob gets the measurement results sequence M_b

Step 5: Alice generates n pairs of random bits $K_a = \{(k_0^1, k_1^1), (k_0^2, k_1^2), \cdots, (k_0^n, k_1^n)\}$ as key. Then Alice records the indices i_a^k where the measurement results satisfy

$$(M_{a1}^{i_a^k}, M_{a2}^{i_a^k}) = (b_0 \oplus k_0^{i_a^k}, b_1 \oplus k_1^{i_a^k}) \tag{5}$$

and arranges all the qualified indices into sequence $I_a = \{i_a^1, i_a^2, \cdots\}$. Then Alice sends I_a to Bob with the confidential channel.

Step 6: Bob records the indices i_b^k where the measurement results satisfy

$$M_{b1}^{i_a^k} = s \tag{6}$$

and arrange the indices as sequence I_b. After receiving I_a, Bob computes the intersection of I_a and I_b. Then Bob randomly sends an index $i_b \in I_a \cap I_b$ and sends i_b to Alice with confidential channel.

Step 7: Alice sends the key pair $(k_0^{i_b}, k_1^{i_b})$ to Bob with the confidential channel.

Step 8: Once receiving the key pair, Bob obtains the result b_s by decrypting the measurement result according to

$$b_s = M_{b2}^{i_b} \oplus k_s^{i_b}. \tag{7}$$

Table 1: The scheme for quantum oblivious transfer.

3 Correctness and Security Analysis of QOT

3.1 Correctness

Theorem 1 (Correctness). *Suppose all the participants (Alice, Bob and Trent) follow the QOT protocol honestly and the QOT protocol finishes normally, then Bob would get the result b_s.*

Proof. From the description of the QOT protocol in Table 1, we can get:

$$M_{b2}^{i_b} = \begin{cases} M_{a1}^{i_b}, & M_{b2}^{i_b} = 0 \\ M_{a2}^{i_b}, & M_{b2}^{i_b} = 1 \end{cases} \tag{8}$$

Therefore we have:

$$M_{b2}^{i_b} \oplus k_s^{i_b} = b_s \oplus k_s^{i_b} \oplus k_s^{i_b} = b_s. \tag{9}$$

□

Theorem 2 (Success rate). *Assuming all the participants in the protocol are honest, the protocol can finish in a probability of* $1 - \left(\frac{7}{8}\right)^n$.

Proof.

$$\begin{aligned} P(1) =& P(I_a \cap I_b \neq \varnothing) \\ &= 1 - P(I_a \cap I_b = \varnothing) \\ &= 1 - \prod_{k=1}^{n} P\left((M_{a1}^{i_a^k}, M_{a2}^{i_a^k}) \neq (b_0 \oplus k_0^{i_a^k}, b_1 \oplus k_1^{i_a^k}) \vee M_{b1}^{i_a^k} \neq s \right) \\ &= 1 - \prod_{k=1}^{n} \left(1 - P\left((M_{a1}^{i_a^k}, M_{a2}^{i_a^k}) = (b_0 \oplus k_0^{i_a^k}, b_1 \oplus k_1^{i_a^k}) \wedge M_{b1}^{i_a^k} = s \right) \right) \\ &= 1 - \prod_{k=1}^{n} \left(1 - \frac{1}{4} \cdot \frac{1}{2} \right) \\ &= 1 - \left(\frac{7}{8} \right)^n \end{aligned} \quad (10)$$

□

Remark that when a large n is adopted, our QOT protocol can finish normally with a probability close to 1.

3.2 Security

3.2.1 Outside Attack

The possible eavesdropper can be detected by checking the measurement result.

Suppose Eve is an eavesdropper. Since Eve knows nothing about the position and the polarization of the decoy particles, Eve cannot select the measurement bases corresponding to the particles, and wrong measurement bases can be chosen with a probability of $\frac{1}{2}$. If Eve measures a single decoy particle with the wrong basis, the eavesdropping can be detected with a probability of $\frac{1}{4}$, and the probability becomes $\frac{3}{4}$ when Trent is measuring a pair of decoy particles. If m pairs of decoy particles are measured, Eve will be detected with a probability of $1 - \left(\frac{1}{4}\right)^m$, which tends to be 1 when m is large. Furthermore, as neither Alice nor Bob sends private information before checking the decoy particles, and the states Trent preparing contain no private information, no private information of Bob's or Alice's can leak through Eve's measurement.

3.2.2 Participant Attack

For the attacks from participants, Trent's attempt to steal the information is considered first. Trent may be dishonest and deviate from the protocol by preparing states entangled with Trent's personal state $|f(b_1, b_0, s)\rangle$:

$$\sum_{b_0=0}^{1} \sum_{b_1=0}^{1} \sum_{s=0}^{1} |b_1, b_0, s, b_s\rangle \otimes |f(b_1, b_0, s)\rangle, \quad (11)$$

where f is the function of the first four bits. In this case, Trent might get Alice or Bob's measurement results by measuring $|f(b_1, b_0, s)\rangle$. However, Trent can only get the exact value of b_0, b_1, or s if and only if Trent knows the value of indices I_a (I_b). Since the transmission of states in QOT protocol is assumed to use strictly confidential channels such as a one-time pad with the quantum key distribution, Trent can obtain no information about Alice or Bob's private information, but only a sequence of random bits with such entanglement attacks.

Then we consider the attack from Alice or Bob. If either party tries to intercept the quantum communication between the other party and Trent, this will be equivalent to an outside attack that is demonstrated to be infeasible. The only information comes from I_b or i_a. However, for $c \in \{0,1\}$, $P(s = c|I_b) = P(b_{s-1} = c|I_b, i_a) = \frac{1}{2}$ always holds and no unnecessary information is leaked from I_b or i_a.

3.2.3 The rationality of non-collusive setting

In the protocol, Trent is required not to collude with Alice or Bob to ensure the security. In classical settings, Trent can always distribute pseudo-random key pairs which follow the oblivious transfer relationship to Alice and Bob, and share the pseudo-random keys to one party (say Alice) in advance. Based on the pseudo-random keys M_{b2} and the Bob's response i_b in Step 6, Alice can then learn the confidential selection bit s of Bob's.

However, here we show a quantum-based case that such collusion can be detected.

First, notice that Trent only emits signals with public channels and unconditionally secure quantum channels. Therefore, it can be rationally supposed that all physical channels of Trent's are public to both Alice and Bob. Either party can intercept the communication at any time without affecting the security. Fig. 1 shows a diagram for such a case, where Alice or Bob can intercept other party's communication with Trent using a controlled reflector.

Let's assume that Alice tries to intercept the transmission of particles from Trent to Bob in Step 4. At any moment in Step 4, Alice can intercept the particles Trent sending to Bob with the reflector and send it to itself. Then Alice will receive the particles Trent sending to Bob and measure the state $|\psi_{ab}\rangle$. If Alice is dishonest and does not claim all the particles it intercepts before Bob receives the positions of the decoy particles, such an action will be equivalent to eavesdropping and will be detected by Bob. Therefore such an interception does not affect the security of Trent's or Bob's.

After getting the whole state $|\psi_{ab}\rangle$, Alice can measure the whole state with POVM and check if $|\psi_{ab}\rangle$ follows Eq. (1). In Step 6, Alice records the numbers of states that are neither claimed to be decoy particles nor following Eq. (1). If the proportion of such states is over a certain threshold determined by noise, Alice will find that Trent is not honestly sending the pre-agreed states.

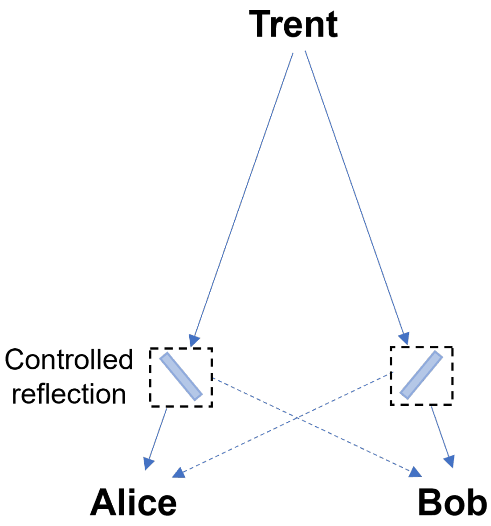

Figure 1: A schematic diagram for the case where Alice or Bob can intercept the other party's communication with Trent and sent it to itself at any time with a controlled reflector.

4 Noise Analysis

As implied above, our QOT protocol does not deal with the quantum noise, but honestly passes errors of the protocol to the next step. The error of the QOT is handled by the DNN model based on its noise tolerance.

The variance of a numerical computation with an n-shot noiseless binary computation is given as

$$\begin{aligned}\sigma^2 &= \mathbb{E}\left(\frac{1}{n}\sum_{i=1}^{n} x_i^2\right) - P^2 \\ &= \frac{1}{n^2}\left(\sum_{i=1}^{n}\mathbb{E}(x_i^2) + \sum_{i\neq j}\mathbb{E}(x_i x_j)\right) - P^2 \\ &= \frac{1}{n^2}\big((n^2-n)P^2 + nP\big) - P^2 \\ &= \frac{P-P^2}{n},\end{aligned} \tag{12}$$

where P is the expected value of computation, and x_i is the random outcome of a single binary computation. Especially, when only one shot binary computation is applied, the variance follows

$$\sigma^2 = P - P^2. \tag{13}$$

According to Equations (11) to (13) in the main text, the error rate λ of a single binary computation in our method based on m noisy QOT operations is

$$\lambda = \frac{1}{2} - \frac{1}{2}\left(1 - 2\epsilon\right)^m, \tag{14}$$

where ϵ is the error rate of a single operation. Denoting the correct result as R, the unbiased computing result r is corrected with

$$r = \frac{p - \lambda}{1 - 2\lambda}, \tag{15}$$

where $p = \frac{1}{n}\sum_{i=1}^{n} x_i, \mathbb{E}\left(p\right) = P$ is the mean of the result of binary computations, and we have

$$\mathbb{E}\left(r\right) = R. \tag{16}$$

The variance of the corrected result is given by

$$\begin{aligned} \sigma^2 &= \mathbb{E}\left(\left(\frac{p-\lambda}{1-2\lambda}\right)^2\right) - \mathbb{E}(r)^2 \\ &= \frac{\mathbb{E}\left(\left(\frac{1}{n}\sum_{i=1}^{n} x_i\right)^2\right) - 2\lambda\mathbb{E}\left(\frac{1}{n}\sum_{i=1}^{n} x_i\right) + \lambda^2}{\left(1-2\lambda\right)^2} - \frac{P^2 - 2\lambda P + \lambda^2}{\left(1-2\lambda\right)^2} \\ &= \frac{P - P^2}{n\left(1-2\lambda\right)^2} \\ &= \frac{(1-\lambda)R + \lambda(1-R) - \left((1-\lambda)R + \lambda(1-R)\right)^2}{n\left(1-2\lambda\right)^2}. \end{aligned} \tag{17}$$

In summary, the noise amplifies the variance of computation by $\frac{1}{(1-2\lambda)^2}$ times.

Supposing the noise tolerance of the neural network is σ_{max}, which means the accuracy of the neural network remains at an acceptable level when the input is added with a noise of variance σ_{max}, and the maximum expected result is M, the noise of the protocol must obey

$$\frac{(1-\lambda)M + \lambda(1-M) - \left((1-\lambda)M + \lambda(1-M)\right)^2}{n\left(1-2\lambda\right)^2} < \sigma_{max}^2. \tag{18}$$

Considering that he error rate λ is usually lower than $\frac{1}{2}$, when $M < \frac{1}{2}$, the error rate is bounded by:

$$\lambda < \frac{\sqrt{4n\sigma_{max}^2 + \left(1-2M\right)^2} + 4n\sigma_{max}^2 + 4M^2 - 4M + 1}{8n\sigma_{max}^2 + 2\left(1-2M\right)^2}, \tag{19}$$

and a more relaxed upper bound is

$$\lambda < \frac{1}{2} - \frac{1}{4\sqrt{n}\sigma_{max}}. \tag{20}$$
